# Mechanobiology of Matricellular Proteins in Bladder Cancer: A Narrative Review and Bioinformatics Analysis

**DOI:** 10.3390/biom16081191

**Published:** 2026-08-14

**Authors:** Alim Turgaliyev, Roman Konovalov, Anton Borissenko, Dieter Riethmacher

**Affiliations:** 1 Department of Biomedical Sciences, School of Medicine, Nazarbayev University, Astana 010000, Kazakhstan; alim.turgaliyev@nu.edu.kz (A.T.);; 2 Human Development and Health, Faculty of Medicine, University of Southampton, Southampton SO16 6YD, UK

**Keywords:** matricellular proteins, bladder cancer, matrix stiffness, extracellular matrix remodeling, mechanotransduction loop, periostin, thrombospondin, SPARC

## Abstract

The extracellular matrix (ECM) in cancer differs from healthy tissue in structure, composition, and mechanical properties. Matricellular proteins (MCPs) play important roles in shaping ECM architecture during tissue remodeling. This narrative review, combined with a bioinformatics analysis, examines six major MCP families—Fasciclins, Tenascins, Thrombospondins, Small Leucine-Rich Proteoglycans, the SPARC family, and the CCN family—through a mechanobiological lens in bladder cancer. It summarizes current knowledge on the mechanical regulation of MCP expression, their effects on matrix stiffness, and their contributions to bladder cancer progression. Analyses of public datasets reveal that stromal cells are the predominant source of MCPs in the tumor microenvironment. Furthermore, mechanical upregulation and involvement in the formation of stiff ECM highlight MCPs as important players in a mechanotransduction feedback loop. While most MCPs exert pro-tumorigenic effects on bladder cancer cells, several display context-dependent or anti-tumorigenic activities. Existing studies have primarily focused on the isolated effects of MCPs on bladder cancer cell lines in two-dimensional systems or simple subcutaneous xenograft models. Both approaches fail to capture the context-dependent nature of MCPs and their involvement in ECM formation. These findings underscore the need for future studies to investigate the complex effects of MCPs on bladder cancer progression.

## 1. Introduction

Bladder cancer is a major health problem worldwide and is one of the most common cancers affecting the urinary system [1]. According to The Global Cancer Observatory, bladder cancer ranks as the 9th most common cancer globally in 2022 and the 6th most common cancer in males [2]. From a health economics perspective, the mean lifetime costs of bladder cancer range from $117,503 to $190,996 across stages [3]. Clinically, outcomes are further complicated by the fact that residual disease remains in about 31% of patients after initial surgery [4]. In 2022, 220,349 deaths occurred due to bladder cancer [2]. These clinical, social, and economic burdens underscore the urgent need to better understand the mechanisms driving tumor invasion, metastasis, and overall aggressiveness, which is vital for developing new and more effective treatment strategies for bladder cancer.

The extracellular matrix (ECM) is far more than a passive scaffold for cells. It participates in a dynamic, reciprocal interaction with cells in which cells continuously remodel the ECM while the ECM, in turn, modulates cellular behavior, signaling, and function [5]. Understanding the logic of ECM organization and remodeling is essential for evaluating its role in both healthy and diseased conditions, such as cancer.

ECM has two main types of organization: basement membrane (a sheet-like structure) and the interstitial matrix (a fibrous network) [6]. The fibrous components of the ECM, primarily collagen, elastin, and fibronectin, assemble hierarchically from protofibrils to fibrils (≈100 nm), then into fibers (≈1000 nm), and finally several fibers and substances between fibers form the ECM (≈1,000,000 nm) [7]. Collagen fibers are the major component of the ECM, and appear as mature (densely packed, larger fibers) and immature fibers (loosely packed, smaller fibers) under picrosirius red staining with polarized light microscopy [8,9]. Remodeling maintains equilibrium between constructive synthesis and assembly of new components and destructive proteolytic degradation. This balance governs ECM architecture.

In cancer, the ECM structure, composition, and mechanical properties differ substantially from those of normal tissue [10]. In normal conditions, fibroblasts are the major source of ECM components [11]. However, the tumor matrix can be synthesized not only by cancer-associated fibroblasts (CAFs) but also by cancer cells themselves [10]. Excessive deposition of ECM components stiffens the matrix, promoting epithelial-mesenchymal transition, invasion, and metastasis while boosting resistance to drugs and immunotherapy [12]. ECM in muscle-invasive bladder cancer displays increased fibril density and alignment, reduced fibril width, and greater vascularization than normal ECM [13]. Stiffness and linearization of the ECM both correlate with and drive bladder cancer progression [13,14,15,16,17,18,19,20].

Matricellular proteins (MCPs) are a group of non-structural ECM proteins whose functions are mediated by interactions with matrix proteins, cell-surface receptors, and molecules such as cytokines and proteases [21]. MCPs are highly expressed during development, exhibit low expression in healthy adult tissues, are transiently re-expressed during tissue repair, and are usually elevated in conditions such as cancer and fibrosis [22,23,24]. Although animals lacking MCPs are generally viable and appear normal or exhibit only subtle phenotypic abnormalities, gene deletions frequently result in defects in collagen architecture, underscoring the critical role of MCPs in shaping structural elements [22]. MCPs are also key contributors to the formation of the tumor microenvironment and its biomechanical properties [24]. This review focuses on six protein families conventionally classified as matricellular: Fasciclins (POSTN, TGFBI), Tenascins (TNC, TNXB, TNR, TNN), the SPARC family (SPARC, SPARCL1, SMOC1, SMOC2, SPOCK1–3), the CCN family (CCN1–6), Thrombospondins (THBS1–4, COMP), and Small Leucine-Rich Proteoglycans (DCN, BGN, LUM, ASPN).

Mechanobiology is a field that focuses on how physical forces and mechanical properties influence cells and tissues [25]. Mechanical cues arising from solid stress within expanding tumors and elevated ECM stiffness can activate cancer and resident cells. This activation forms a self-reinforcing positive feedback loop where increased matrix rigidity promotes nuclear translocation of mechanosensitive transcription factors, which in turn drive ECM remodeling and further matrix stiffening, thereby perpetuating the activated, pro-fibrotic state [26,27,28]. A range of mechanosensors, including Piezo1, TRPV4, and integrins, activate multiple intracellular pathways such as TGF-β–SMAD2/3, Ras/ERK, YAP/TAZ, RhoA/ROCK, PI3K/AKT, and Wnt/β-catenin [29]. Since most MCPs lie downstream of these pathways, mechanical stimulation is expected to upregulate their expression. Mechanical induction, together with the established roles of MCPs in tissue remodeling, positions these proteins as potentially important mediators of the mechanotransduction loop. MCPs can sustain this pro-fibrotic loop both indirectly, by altering matrix stiffness, and directly, through interactions with surface receptors such as integrins.

This article investigates matricellular proteins from a mechanobiological perspective, with particular emphasis on bladder cancer. It synthesizes current knowledge on how MCPs respond to mechanical stimulation, their effects on matrix stiffness, and the roles these proteins play in bladder cancer progression and the CAF-centered mechanotransduction loop, while also highlighting their potential as biofluid markers and discussing methods for capturing their complex roles.

## 2. Mechanotransduction of Matricellular Proteins

**Figure 1:**
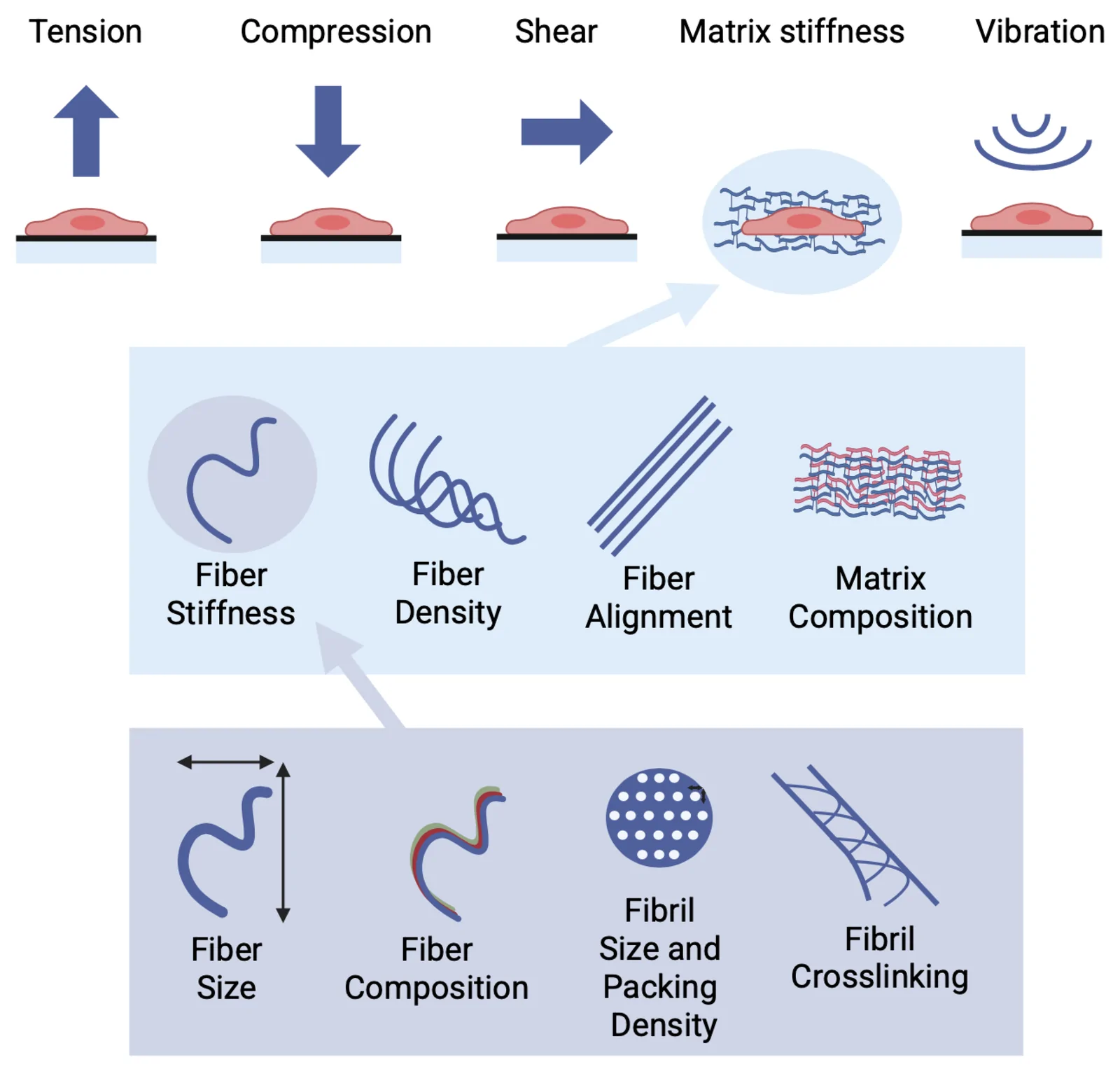
Mechanical stimuli classification and ECM stiffness factors. Created in BioRender. Turgaliyev, A. (2026) https://BioRender.com/xv6jf29, accessed on 2 August 2026.

Mechanotransduction is the process by which cells sense and convert mechanical stimuli into biochemical and behavioral responses. These stimuli fall into five broad categories (Figure 1): tension (uniaxial or multiaxial stretch, membrane tension, and negative pressure); compression (solid compression, positive pressure, and hypergravity); shear stress (solid or fluid); changes in substrate/scaffold/matrix stiffness; and vibration.

Most matricellular proteins are upregulated by mechanical stimulation (Table 1) [30,31,32,33,34,35,36,37,38,39,40,41,42,43,44,45,46,47,48,49,50,51,52,53,54,55,56,57,58,59,60,61,62,63,64,65,66,67,68,69,70,71,72,73,74,75,76,77,78,79,80]. The fact that multiple distinct stimuli can upregulate the same MCPs suggests shared signaling pathways mediate these responses. However, upregulation of MCPs after mechanical stimulation is not universal. Differential expression of MCPs may result from several factors, including cell- and tissue-specific responses, the requirement for an optimal intensity [35,37,49] and duration [33], opposing effects of laminar versus disturbed flow [45,57], different responses in normal versus diseased cells [74,76], and distinct behaviors of differentiated (mature) versus undifferentiated (immature) cells [54]. Other environmental factors and pharmacological agents may also modulate MCPs expression in response to mechanical stimulation. In addition, temporal patterns must be considered, as MCPs expression frequently rises transiently before subsequently declining [51,52,70].

These mechanotransduction principles of MCP expression are especially relevant in the bladder, a heterogeneous structure composed of several layers with distinct ECM architectures [13,81] and stiffness [82,83]. This layered organization naturally leads to consideration of spatial patterns of MCP expression, in which the same cells across different layers can produce different MCPs or express them at different levels in response to their local mechanical microenvironment.

**Figure 2:**
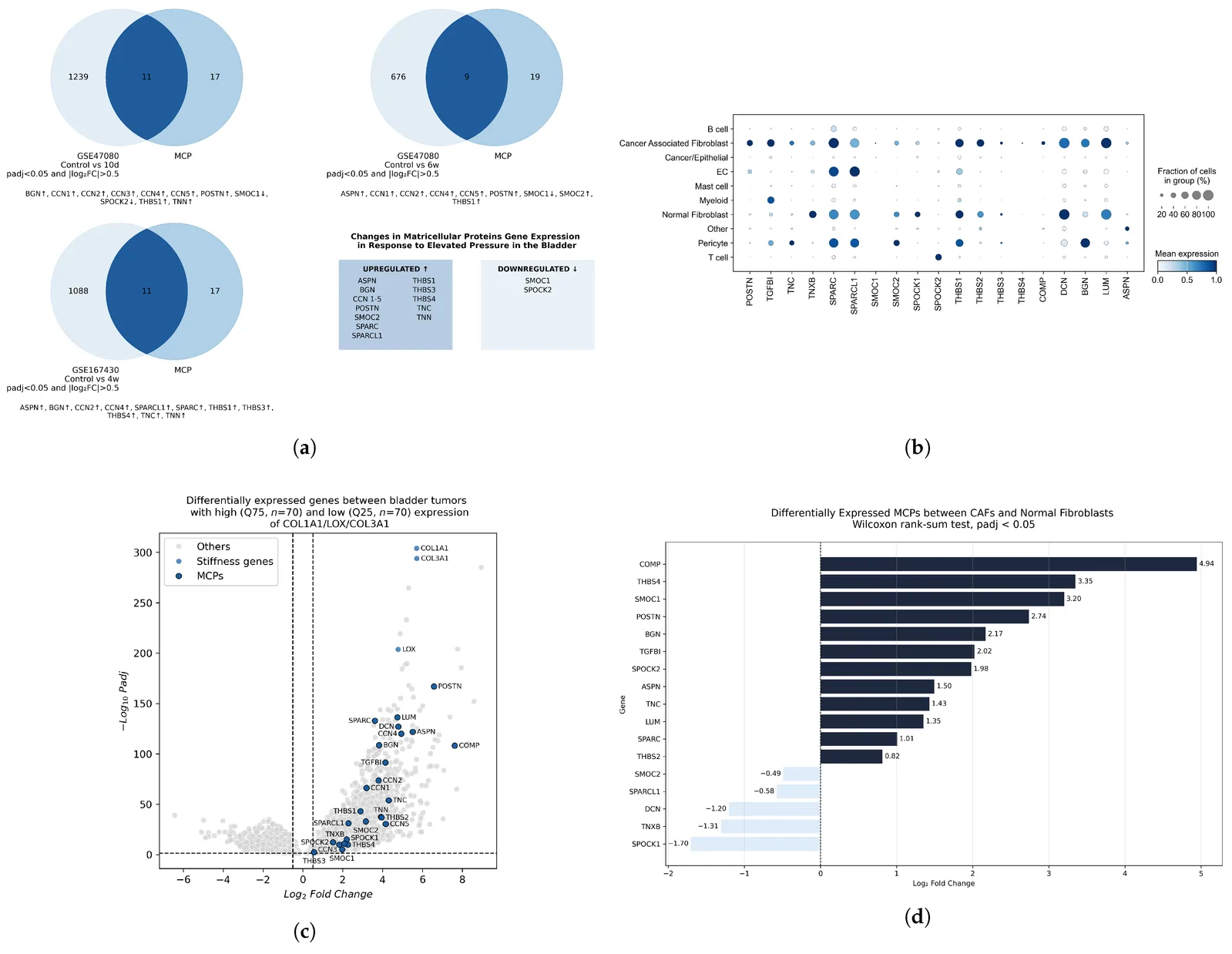

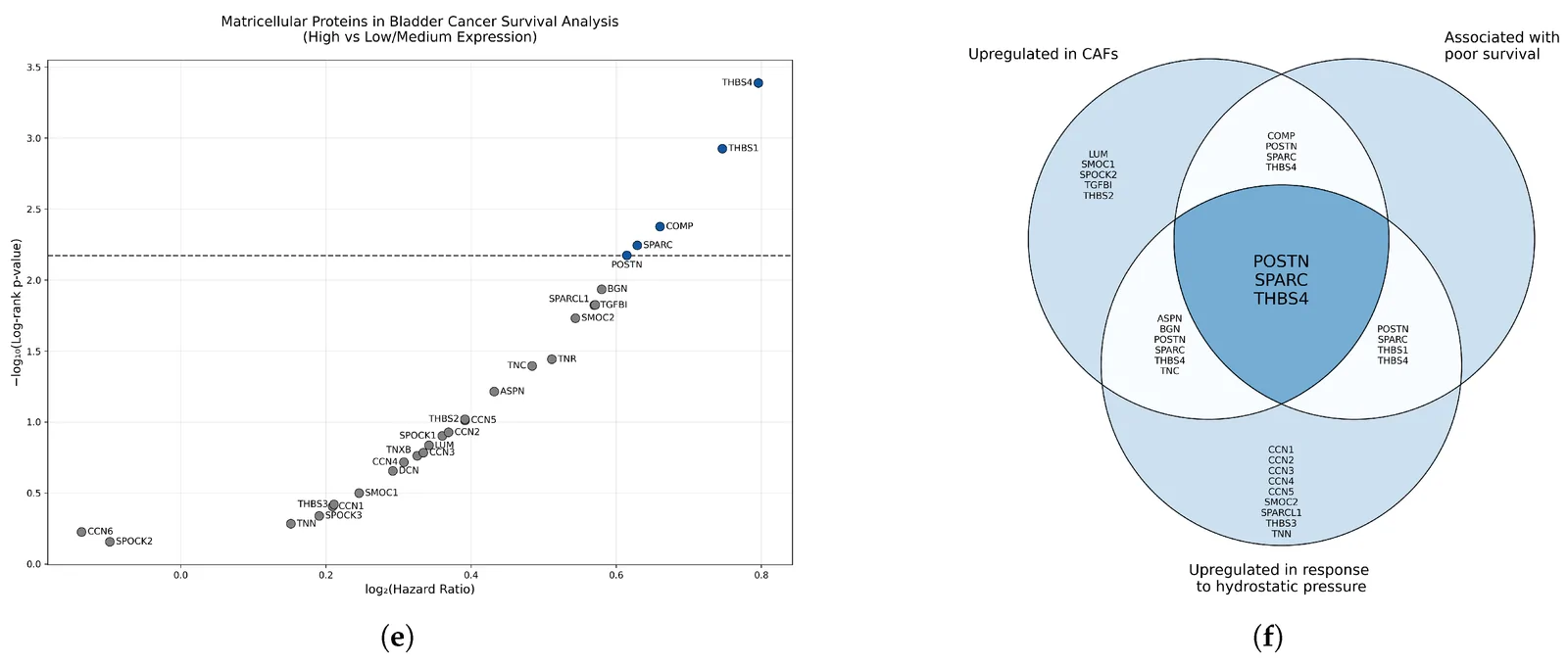
Bioinformatic characterization of matricellular proteins in bladder cancer. (**a**) Differential expression analysis of MCPs in rat bladder partial outlet obstruction datasets. (**b**) MCP expression profiles in single-cell RNA-seq (SCP3121) of normal and cancerous bladder tissue. (**c**) Differential expression analysis between stiffness-stratified bladder tumors in TCGA-BLCA. (**d**) Differentially expressed MCPs in cancer-associated fibroblasts (CAFs) versus normal fibroblasts in SCP3121 dataset. (**e**) MCPs in Bladder Cancer Survival Analysis in TCGA BLCA cohort. (**f**) Overlap of MCPs across analyses (Venn diagram). Detailed information about materials and methods can be found in Appendix A.

At the same time, the bladder wall experiences continuous physiological mechanical loading, primarily from cyclic stretch and intravesical pressure changes during filling and voiding. Partial bladder outlet obstruction (BOO) increases resistance to urine outflow, necessitating stronger detrusor contractions and higher voiding pressures. Consequently, greater mechanical forces are exerted on the bladder wall, driving fibroblast-to-myofibroblast transition and excessive production of ECM components [84]. Differential expression analysis of publicly available rat partial BOO datasets [85,86] identified 16 upregulated (ASPN, BGN, CCN1-5, POSTN, SMOC2, SPARC, SPARCL1, THBS1, THBS3, THBS4, TNC, TNN) and 2 downregulated (SMOC1, SPOCK2) matricellular proteins (Figure 2a). These proteins may represent a bladder tissue-specific MCP signature of the adaptive (and potentially maladaptive) response to elevated mechanical stimulation.

## 3. Matrix Stiffness and Matricellular Proteins

Interstitial ECM stiffness is governed by several key contributors (Figure 1), including matrix composition, fiber density, fiber alignment, and fiber stiffness (which, in turn, depends on fiber size, fiber composition, fibril size and packing density, and fibril crosslinking). Consequently, any alterations in collagen fiber maturation, density, alignment, or crosslinking can change ECM rigidity.

**Figure A1:**
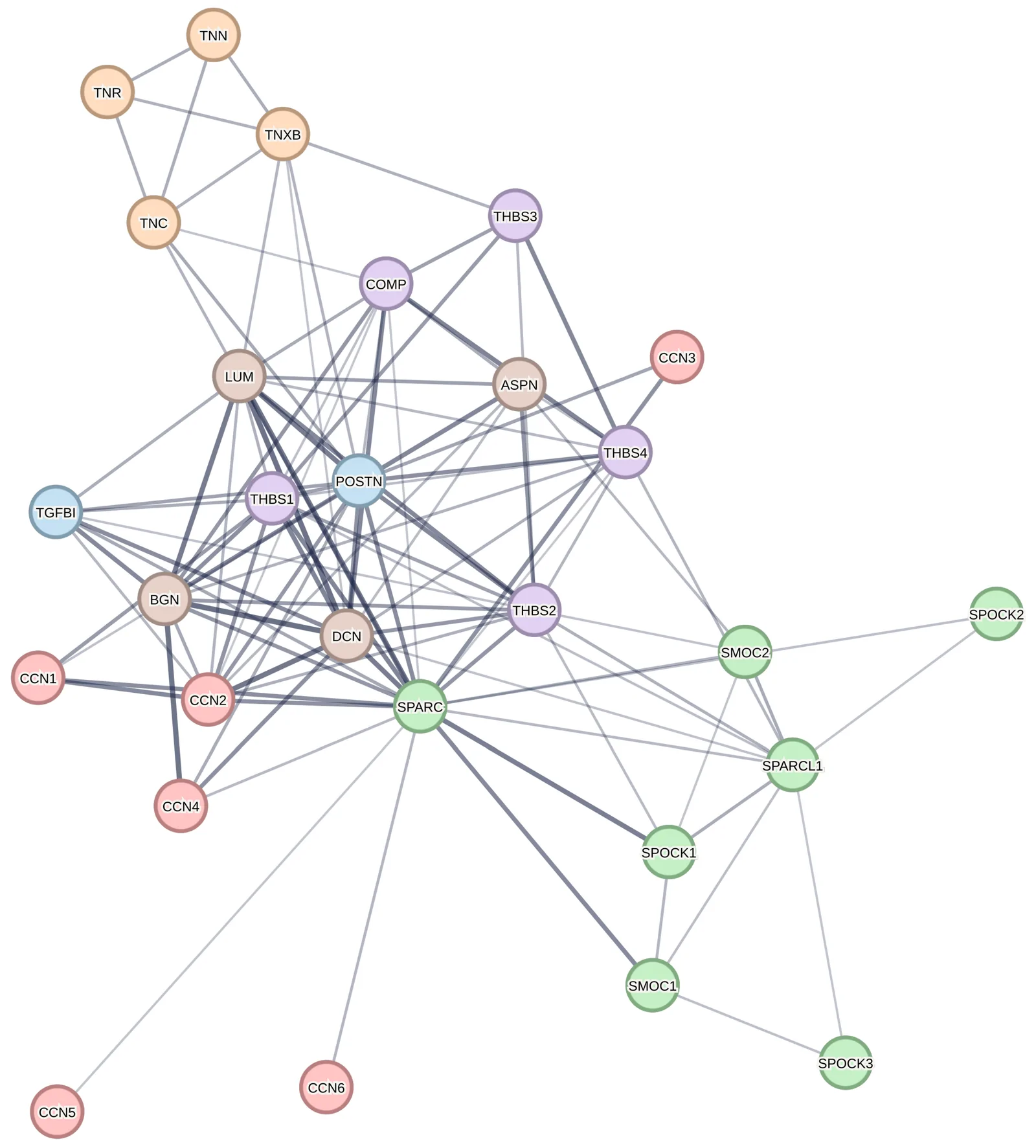
Protein-protein interaction network among matricellular proteins. Detailed information about materials and methods can be found in Appendix A.1.

Changes in matricellular proteins alter collagen fiber organization, as documented in numerous studies (Table 2) [87,88,89,90,91,92,93,94,95,96,97,98,99,100,101,102,103,104,105,106,107,108,109,110,111,112,113,114,115,116,117,118,119,120,121,122,123,124,125,126,127,128,129,130,131,132,133,134,135,136,137,138,139,140]. Because organs have distinct compositions and structures, it is not surprising that knocking out some MCPs leads to distinct changes in collagen fibers across organs [128,134]. Considering that MCPs interact extensively not only with cells and other ECM components but also with each other (Figure A1), it is not clear if effects on ECM structure are governed by specific MCPs alone or in combination with other MCPs. For example, SPARCL1 leads to downregulation of DCN in the skin [104], CCN5 inhibits the effect of fiber linearization by CCN4 [116], and ASPN deficiency is associated with doubled DCN and BGN levels in the skin [138]. It also remains unclear whether the effects of MCPs on collagen fiber organization are driven by direct interactions or arise indirectly through impaired cellular recruitment [90,100,110].

Importantly, alterations in matrix stiffness are not solely determined by changes in collagen fiber quantity or organization. Specific MCPs, such as TNXB, can directly influence the material mechanical properties [98].

Notably, while the influence of MCPs on ECM architecture and mechanical properties has been extensively characterized in tissues such as skin, tendon, cornea, and heart, the effects of MCPs on the organization and stiffness of the bladder ECM remain largely unexplored. These insights could prove valuable not only for identifying potential therapeutic targets against pathological remodeling in cancer and fibrosis, but also for applications in bladder tissue engineering and regenerative medicine.

## 4. Matricellular Proteins in Bladder Cancer

### 4.1. Expression of Matricellular Proteins in Bladder Cancer

**Figure A2:**
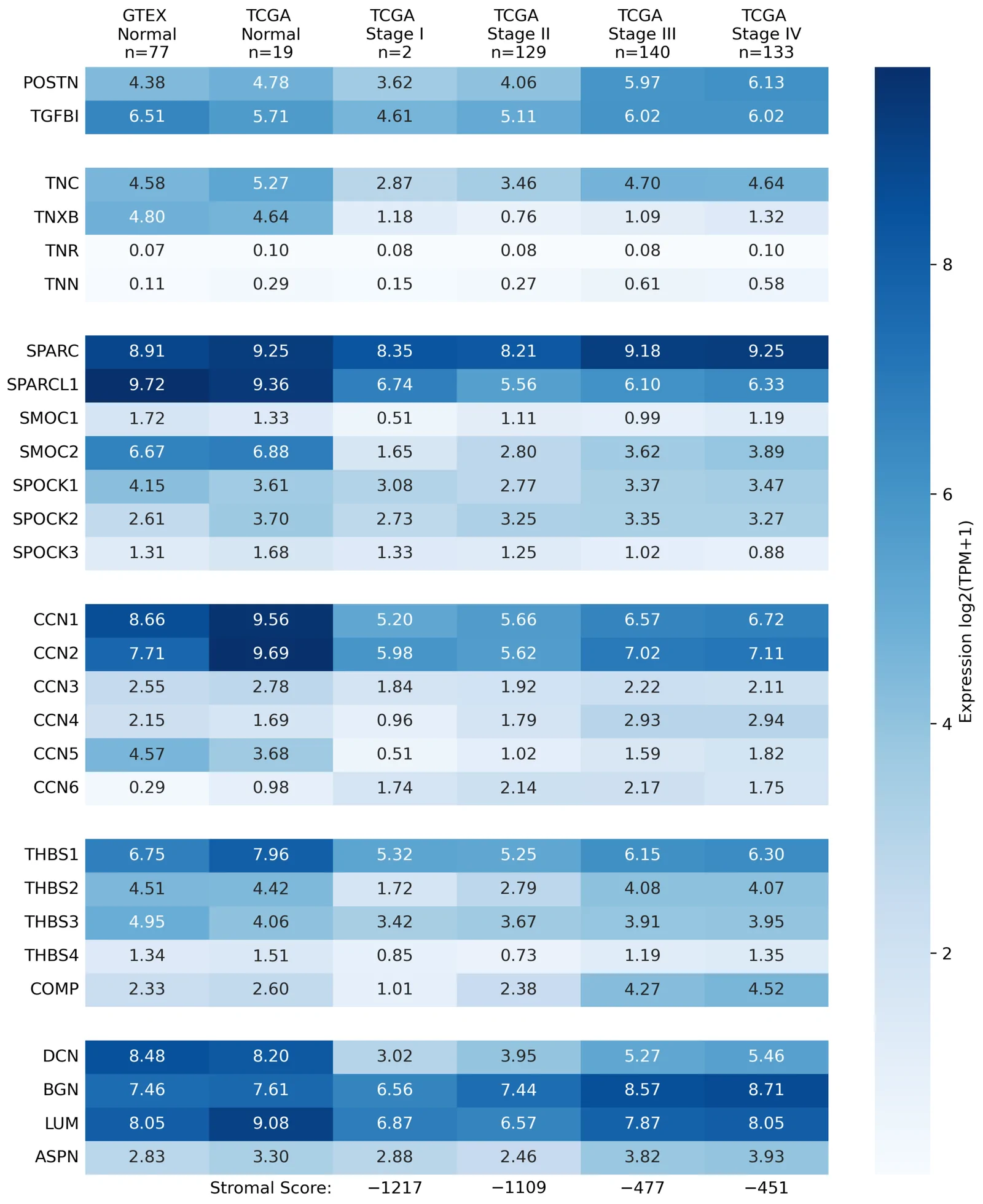
Matricellular protein gene expression in normal bladder tissue (GTEx) and bladder urothelial carcinoma (TCGA-BLCA) across pathological stages. Detailed information about materials and methods can be found in Appendix A.2.

**Figure A3:**
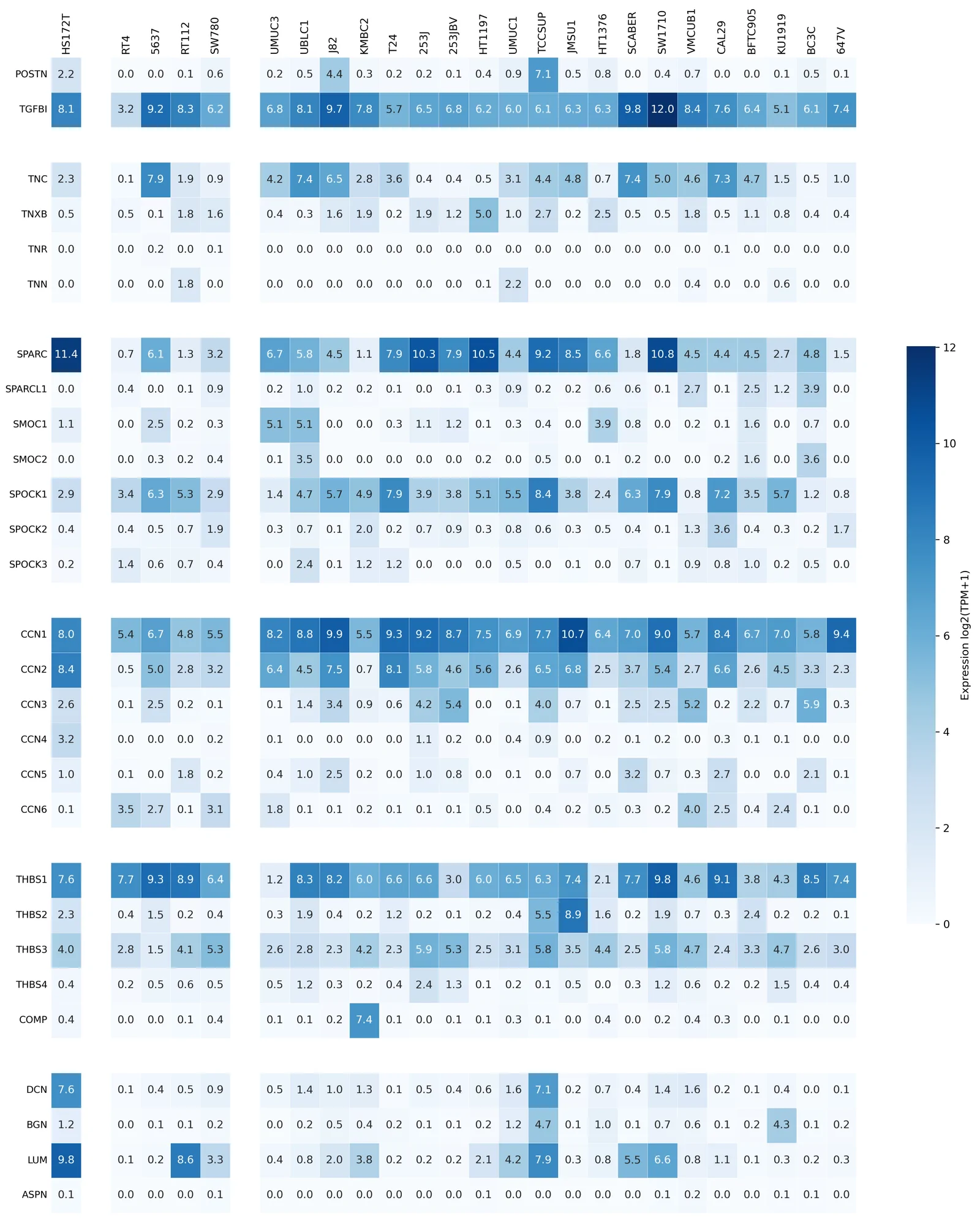
Matricellular protein gene expression in bladder cancer cell lines (CCLE). Detailed information about materials and methods can be found in Appendix A.4.

MCPs expression in the TCGA-BLCA dataset follows a U-shaped trajectory across pathological stages (Figure A2), most likely due to differences in the stromal cells that were sequenced. Consistent with this interpretation, the majority of bladder cancer cell lines show low transcripts per million (TPM) values for most MCPs (Figure A3). The notable exceptions are TGFBI, TNC, SPARC, SPOCK1, CCN1, CCN2, THBS1, and THBS3. Strikingly, the HS172T line, originally classified as a bladder cancer cell line but suggested to be a fibroblast line [141], exhibits a wider repertoire of MCPs. However, some bladder cancer cell lines, such as J82 and TCCSUP, also display broader MCP expression patterns, implying that while stromal cells are the dominant source, cancer cells also contribute to MCP production.

Single-cell RNA sequencing provides direct cellular resolution and confirms the bulk-level observations. CAFs and normal fibroblasts emerge as the dominant MCP-expressing populations, followed by pericytes and endothelial cells, whereas epithelial and cancer cells contribute minimally (Figure 2b). Within the immune compartment, SPOCK2 stands out for its high expression in T cells, suggesting that this MCP may participate in immune modulation. Together, these data position stromal cells, especially CAFs, as the principal source of MCPs in the bladder tumor microenvironment.

### 4.2. Matricellular Proteins and CAF-Centered Mechanotransduction Loop in Bladder Cancer

CAFs are a heterogeneous population of stromal non-malignant cells with multiple origins, including a transition of resident fibroblasts [142]. Although there is evidence that certain CAF subsets restrain tumor growth, such that their depletion accelerates progression and reduces survival [143], CAFs are generally considered pro-tumorigenic. Historically, CAFs were classified into inflammatory CAFs (iCAFs) and myofibroblast CAFs (myCAFs), with myCAFs located closer to the tumor mass and iCAFs more distal [144]. Similar CAF subtypes have also been identified in bladder cancer [145]. Recent evidence suggests the presence of additional subtypes, such as antigen-presenting CAFs (aCAFs) and others [146]. Despite these advances, a standardized consensus classification for CAFs and their function has yet to be established.

The expression of MCPs could serve as a distinguishing feature for separating specific CAF subpopulations in single-cell RNA sequencing data. For instance, in the two-type bladder cancer CAF classification, DCN and LUM are expressed at higher levels in iCAFs compared to myCAFs [145]. In a pan-cancer analysis of CAFs, MCPs were used, among other markers, to distinguish CAF subpopulations. For example, THBS2 and POSTN were used as markers of myCAFs, DCN and TNXB as markers of iCAFs, TGFBI as a marker of hypoxia-associated CAFs, SPARC as a marker shared by both myCAFs and iCAFs, BGN as a marker of both myCAFs and early-activated CAFs, and SPARCL1 as a marker of early-activated CAFs [147].

Beyond their utility as separation markers, MCPs’ expressions are also linked to the mechanisms that generate and sustain CAFs. CAF formation can be driven by biochemical factors as well as mechanical forces.

Exosomes secreted by bladder cancer cells induce the differentiation of fibroblasts into CAFs primarily through TGF-β signaling [148]. Notably, both MCPs and TGF-β [32,35,39,55,60,69,79] are upregulated in response to mechanical stimulation, suggesting that they may converge on the same TGF-β-dependent pathway to promote CAF formation. Moreover, most MCPs have a well-established interaction with TGF-β.

One key mechanical cue is matrix stiffness, which contributes to the development and maintenance of CAFs [27]. COL1A1, LOX, and COL3A1 genes are associated with matrix stiffness [149]. In urothelial bladder carcinoma, MCPs expression is upregulated in samples exhibiting elevated levels of these stiffness-related genes (Figure 2c).

Moreover, CAFs and cancer cells interact mechanically, not only through matrix stiffness. CAFs form a capsule that envelops cancer cells and actively compresses them [150]. At the same time, actively proliferating cancer cells generate solid stress on neighboring CAFs. These forces can upregulate the secretion of mechanoresponsive proteins, such as TGF-β and MCPs, in both cancer cells and CAFs, thereby helping to maintain the CAF population.

Of note, in the BBN-induced bladder cancer rat model, ECM linearization occurs during the inflammatory process and precedes cancer atypia [20]. This raises the possibility that CAF-like fibroblasts and the formation of pro-tumorigenic ECM appear before the emergence of cancer cells.

Direct comparison of CAFs versus normal fibroblasts (Figure 2d) revealed 12 upregulated and 5 downregulated MCPs in bladder cancer CAFs. Interestingly, 4 upregulated MCPs (THBS4, SPARC, POSTN, COMP) showed statistically significant associations with worse outcomes in survival analysis of bladder urothelial carcinoma patients (high vs low/medium expression) (Figure 2e). These findings indicate that the CAF-specific upregulation of this subset of MCPs may actively contribute to tumor progression and adverse clinical outcomes.

Collectively, mechanical stimulus-induced upregulation of MCPs and their subsequent effects on matrix stiffness position these proteins as important nodes in a mechanotransduction feedback loop. SPARC, POSTN, and THBS4 are mechanosensitive matricellular proteins in the bladder that are upregulated in CAFs and associated with adverse clinical outcomes in bladder cancer (Figure 2f). These three MCPs are therefore likely important components of the CAF-centered mechanotransduction loop in which matrix stiffness supports the formation and maintenance of CAFs.

### 4.3. Matricellular Proteins as Biofluid Markers in Bladder Cancer

Given the low expression levels of MCPs under normal conditions and their secretory nature, several studies have examined MCPs as markers of bladder cancer in urine and serum. POSTN was elevated in extracellular vesicles isolated from urine of patients with muscle-invasive bladder cancer compared to non-muscle-invasive bladder cancer patients and healthy controls [151]. Similarly, the TGFBI/creatinine ratio was significantly higher in urothelial carcinoma patients than in both hospital and population controls; it was also elevated in de novo cases relative to recurrent cases, in high-grade tumors compared with low-grade tumors and hospital controls, and in muscle-invasive patients compared with non-muscle-invasive patients and hospital controls [152]. TGFBI concentrations can be elevated not only in urine but also in serum, compared with those in patients with benign prostatic hyperplasia and healthy controls [153]. Urinary concentrations of TNC and its domains were higher in patients with bladder cancer and urinary tract infection than in healthy volunteers [154,155]. In serum, BGN and LUM levels were elevated, whereas DCN levels were decreased, in bladder cancer patients relative to controls [156]. Collectively, these findings highlight the potential of MCPs as biofluid markers in bladder cancer. However, large-scale clinical trials are still required to establish their diagnostic or prognostic utility. For example, SPARC was elevated in urine in both primary and recurrent urothelial bladder cancer patients compared with controls, but its diagnostic performance for detecting bladder cancer was insufficient for clinical implementation. Its performance for monitoring tumor relapse and/or progression appeared more promising, but it was affected by hematuria [157].

### 4.4. Role of Matricellular Proteins in Bladder Cancer Progression

Experimental in vitro evidence from bladder cancer cell lines demonstrates that overexpression of MCPs predominantly promotes invasion, migration, and proliferation, while their knockdown suppresses these behaviors, consistent with a broadly protumorigenic functional profile (Table 3) [151,152,158,159,160,161,162,163,164,165,166,167,168,169,170,171,172,173,174,175,176,177,178,179,180]. However, this is not true in all cases. SPARCL1 showed anti-tumorigenic properties [170], while BGN displayed a proliferation-inhibitory effect on bladder cancer cells [178]. Others like POSTN [160], DCN [177], CCN3 [172], CCN4 [173] show alternative splicing and post-translational modification-dependent roles in bladder cancer cells.

At the same time, most in vivo experiments have relied on xenograft models involving subcutaneous injection of human bladder cancer cells into immunodeficient nude mice (Table 4). In the majority of these experiments, the pro- or anti-tumorigenic effects of MCPs observed in vivo mirrored their corresponding in vitro findings, as reflected by increased or decreased tumor weight/size and metastasis relative to controls.

In several in vivo experiments, matricellular proteins such as POSTN and DCN show context-dependent variation in bladder cancer growth and invasion, varying with both the anatomical site of implantation and the host’s immune competence [162,176,177].

UMUC-3 cells transduced with a vector expressing POSTN produced tumors of comparable weight to vector-control cells when injected subcutaneously into BALB/c nude mice. In contrast, orthotopic implantation into the bladder resulted in lower tumor-bearing bladder weights and abolished muscle invasion. This invasion-preventing effect was caused by the formation of thick, edematous lesions that separated the muscle layers from the neoplastic tissue. These lesions were absent after subcutaneous injection, pointing to possible tissue-specific mechanisms [162].

Another context-dependent behavior was observed with DCN. In a subcutaneous xenograft model, T24 cells expressing full-length DCN formed smaller tumors than controls, whereas cells expressing non-glycanated DCN produced markedly larger tumors [177]. Conversely, in a syngeneic model, subcutaneous injection of MB49 cells overexpressing full-length DCN into immunocompetent C57BL/6J mice resulted in larger tumors relative to controls [176]. The more invasive and aggressive MB49-I variant constitutively expresses higher levels of DCN than parental MB49 cells. When DCN was knocked down in MB49-I cells and the cells were introduced orthotopically into the bladder, the weight of tumor-bearing bladders was reduced compared with controls [176]. These divergent outcomes highlight the context-dependent influence of MCPs, arising from differences in host immunocompetence and the molecular form of the MCP.

Beyond their direct effects on bladder cancer cells, MCPs and their context dependence can also shape the behavior of stromal cells within the tumor microenvironment. For example, cancer-associated blood vascular endothelial cells isolated from invasive bladder cancer predominantly express the short isoform of THBS2 rather than the full-length protein. In contrast, healthy blood vascular endothelial cells express both isoforms at comparable levels. Unlike the full-length protein, this short isoform cannot inhibit the proliferation and migration of these cancer-associated endothelial cells, potentially promoting angiogenesis [181]. In parallel, MCPs also play important roles in lymphangiogenesis in bladder cancer.

For example, THBS4 secreted by cancer cells promotes the recruitment of lymphatic endothelial cells and induces VEGF-C-mediated lymphangiogenesis within the tumor microenvironment [182].

One of the most thoroughly studied MCPs in bladder cancer, examined in both cancer cells and the tumor microenvironment, is SPARC. In a BBN-induced bladder cancer model, SPARC-knockout mice showed accelerated progression from inflammation with urothelial atypia and dysplasia through carcinoma in situ to invasive carcinoma. Loss of SPARC was associated with shorter survival, a higher incidence of metastasis, and increased bladder vascularity relative to wild-type controls. SPARC deficiency also produced an inflammatory phenotype in tumor-associated macrophages and fibroblasts. Collectively, these findings indicate that SPARC exerts a predominantly anti-tumorigenic effect in bladder cancer [168]. At the same time, its anti-inflammatory activity implies a potential role in fostering cold tumor phenotypes.

SPARC expression in bladder cancer is predominantly a feature of the stromal compartment rather than the cancer cells themselves. In wild-type mice with BBN-induced bladder cancer, SPARC levels decreased markedly inside the tumor cells as the disease progressed. At the same time, expression remained stable in the surrounding stromal cells [168]. The same pattern is observed in human samples: invasive bladder-cancer cells are negative for SPARC, whereas desmoplastic stromal cells stain strongly [183]. These observations indicate that SPARC is primarily expressed by cancer-associated fibroblasts, particularly myCAFs, which produce high levels of the protein [184]. CAFs isolated from SPARC-knockout mice have a more inflammatory secretome than those from wild-type animals [168], suggesting that loss of SPARC can promote iCAF formation. However, that interpretation needs to be checked in experimental settings. The influence of SPARC on CAF subtype specification may arise not only from biochemical signaling but also via mechanical cues, including matrix stiffness.

## 5. Future Perspectives

Given the complex, context-dependent effects of MCPs on a variety of cell types and on the formation of the tumor microenvironment, appropriate experimental approaches are required to investigate their overall impact on bladder cancer progression. Most existing studies have examined the effects of MCPs on bladder cancer cells in isolation, typically using two-dimensional culture systems. These approaches overlook the reciprocal interactions between cancer cells and stromal components—particularly CAFs—within a mechanically dynamic, three-dimensional ECM. Consequently, the specific contributions of MCPs to cancerous ECM formation and the downstream effect of remodeled ECM on cancer progression remain inadequately defined. To address this gap, experimental designs that include the effect of MCPs on the ECM are needed.

One powerful strategy involves criss-cross orthotopic models in which genetically modified bladder cancer cells (knockout/knockdown or overexpression versus wild-type) are implanted into mice with stromal-targeted alterations (knockout or conditional knockout versus wild-type). This setup allows evaluation of how MCPs in either the cancer or stromal compartment influence tumor progression and matrix remodeling under strictly controlled genetic conditions.

An alternative in vivo approach is to employ well-established chemically induced bladder cancer models, such as those using BBN or MNU [185], in knockout animals with matched wild-type controls, similar to work done with SPARC [168]. Regardless of the in vivo strategy chosen, particular attention should be paid to changes in collagen architecture and ECM stiffness during carcinogenesis when comparing knockout and wild-type animals.

Given that distinct CAF subpopulations differentially express MCPs, future studies should investigate how MCP knockout influences the generation and maintenance of these subtypes. Combining bladder cancer models in wild-type and knockout animals with mechanical characterization of the ECM would be particularly informative, ideally by first quantifying tissue mechanical properties using non-destructive methods such as Brillouin light scattering spectroscopy [83], followed by spatial single-cell RNA sequencing.

In vitro approaches that capture the multiple functions of the ECM can utilize criss-cross three-dimensional co-culture systems in which genetically modified bladder cancer cells are combined with genetically modified CAFs (knockout/knockdown or overexpression versus wild-type) within mechanically tunable ECM scaffolds. These scaffolds can be produced either synthetically or via self-assembly methods [186]. Such models enable direct characterization of reciprocal signaling and force-mediated matrix reorganization under controlled biophysical conditions.

Insights from these approaches will be valuable for understanding how migration tracks form along which cancer cells metastasize and for identifying CAF subpopulations that either restrict or promote disease progression. Collectively, this knowledge may identify specific MCPs as potential therapeutic targets for preventing metastasis and disease progression.

## 6. Conclusions

Mechanotransduction regulates matricellular protein expression across tissues and cells in a context-dependent manner. Although most MCPs are subjected to mechanical stress, their precise regulation is finely tuned by the cellular context, stimulus parameters, and the state of the surrounding environment. Involvement of MCPs in the CAF-centered mechanotransduction feedback loop makes them attractive targets for therapeutic strategies aimed at normalizing pathological ECM remodeling. Further investigation is needed into the role of MCPs in CAF subpopulation differentiation, as well as their clinical significance as biofluid markers for bladder cancer. The majority of MCPs promote tumorigenesis in bladder cancer cells, but several exhibit context-dependent or anti-tumorigenic effects. Most existing studies have focused primarily on isolated effects of MCPs on cancer cells in two-dimensional systems or in heterotopic xenograft models. These approaches fail to capture the reciprocal interactions between cancer and stromal cells within a mechanically dynamic three-dimensional ECM and the bladder-specific context. Future studies should employ advanced models—such as criss-cross orthotopic in vivo models and tunable three-dimensional co-culture platforms—that involve the effects of MCPs on the ECM. Such approaches will be essential for translating mechanistic insights into effective interventions against bladder cancer progression.

## Figures and Tables

**Table 1:** Expression of MCPs in response to mechanical stress.

Protein	Cells/Organ	Mechanical Factor	Expression	Source
POSTN	NOK-SI (human oral keratinocytes)	Stretch	↑	[30]
	R-ASM-580 (rat aortic smooth muscle cells)	Stretch	↑	[31]
	Human ligamentum flavum cells	Fluid shear (disturbed flow)	↑	[32]
	Chicken atrioventricular cushion explants	Fluid shear (laminar flow)	↑	[33]
	Mouse left ventricle	Pressure	↑	[34]
	Human periodontal ligament stem cells	Compression	↑	[35]
	Mouse palatal fibroblasts	Scaffold/substrate stiffness	↑	[36]
TGFBI	Human periodontal ligament cells	Stretch	↑	[37,38]
	Rat chondrocytes	Stretch	↑	[39]
TNC	Human periodontal ligament cells	Stretch	↑	[38]
	Chick embryo skin fibroblasts	Stretch	↑	[40]
	Mouse fibroblasts	Stretch	↑	[41]
	Rat neonatal ventricular myocytes	Stretch	↑	[42]
	Porcine trabecular meshwork cells	Stretch	↑	[43]
	MC3T3-E1 (murine pre-osteoblastic cells)	Fluid shear (disturbed flow)	↑	[44]
	Chicken atrioventricular cushion explants	Fluid shear (laminar flow)	↑	[33]
TNXB	Human umbilical vein endothelial cells	Fluid shear (laminar flow)	↑	[45]
	Human umbilical vein endothelial cells	Fluid shear (disturbed flow)	↔	[45]
TNR	n.d.	n.d.	n.d.	n.d.
TNN	MC3T3-E1 (murine pre-osteoblastic cells)	Fluid shear (disturbed flow)	↑	[44]
SPARC	Human periodontal ligament cells	Stretch	↑	[38]
	Porcine trabecular meshwork cells	Stretch	↑	[43]
	Rat neonatal ventricular myocytes	Stretch	↔	[42]
	Mouse podocytes	Stretch	↑	[46]
	Human bone-marrow-derived mesenchymal stem cells	Stretch	↑	[47]
	R-ASM-580 (rat aortic smooth muscle cells)	Stretch	↑	[31]
SPARCL1	n.d.	n.d.	n.d.	n.d.
SMOC1-2	n.d.	n.d.	n.d.	n.d.
SPOCK1	n.d.	n.d.	n.d.	n.d.
SPOCK2	Human colon epithelial cells	Scaffold/substrate stiffness	↑	[48]
SPOCK3	n.d.	n.d.	n.d.	n.d.
CCN1	Human and murine tenocytes	Stretch	↑	[49]
	MLE-12 cells (mouse alveolar epithelial cells)	Stretch	↑	[50]
	A549 cells (human pulmonary epithelial cells)	Stretch	↑	[51]
	Fetal bovine bladder smooth muscle cells	Stretch	↑	[52]
	Mouse bladder smooth muscle cells	Stretch	↑	[53]
	Undifferentiated human osteoblast cells	Fluid shear (laminar flow)	↓	[54]
	Differentiated human osteoblast cells	Fluid shear (laminar flow)	↑	[54]
	Rat bladder	Pressure	↑	[53]
CCN2	Human periodontal ligament cells	Stretch	↑	[38]
	SW1353 (human chondrosarcoma cell line)	Stretch	↑	[55]
	Human anterior cruciate ligament-derived interface cells	Stretch	↑	[56]
	Human anterior cruciate ligament-derived midsubstance cells	Stretch	↔	[56]
	R-ASM-580 (rat aortic smooth muscle cells)	Stretch	↑	[31]
	Human aortic endothelial cells	Fluid shear (disturbed flow)	↑	[57]
	Human aortic endothelial cells	Fluid shear (laminar flow)	↔	[57]
	Human pancreatic cancer-associated fibroblasts	Scaffold/substrate stiffness	↑	[58]
CCN3	R-ASM-580 (rat aortic smooth muscle cells)	Stretch	↑	[31]
CCN4	Human ligamentum flavum cells	Stretch	↑	[59]
	SV40 large T-antigen immortalized murine proximal tubular epithelial cells	Fluid shear (laminar flow)	↑	[60]
CCN5-6	n.d.	n.d.	n.d.	n.d.
THBS1	Human aortic endothelial cells	Fluid shear (disturbed flow)	↑	[57]
	Human aortic endothelial cells	Fluid shear (laminar flow)	↔	[57]
	Human umbilical vein endothelial cells and human cardiac microvascular endothelial cells	Fluid shear (laminar flow)	↓	[61]
	Human endothelial progenitor cells	Fluid shear (laminar flow)	↓	[62]
	Human pancreatic cancer-associated fibroblasts	Scaffold/substrate stiffness	↑	[58]
	MCF-7 and MDA-MB-231 cells (human breast cancer cell lines)	Scaffold/substrate stiffness	↑	[63]
	MDA-MB-231 cells (human breast cancer cell line)	Scaffold/substrate stiffness	↑	[64]
	R-ASM-580 (rat aortic smooth muscle cells)	Stretch	↑	[31]
	Human keloid fibroblasts	Stretch	↑	[65]
	Human ligamentum flavum cells	Stretch	↑	[66]
	CC-2630 cells (human pulmonary artery endothelial cells)	Stretch	↑	[67]
	Diabetic rat left ventricle	Pressure	↑	[68]
	Mouse bone marrow-derived macrophages	Compression	↑	[69]
THBS2	Rat bone marrow mesenchymal stem cells	Pressure	↑	[70]
	Rat retinal macroglia and neuron cells	Pressure	↑	[71]
THBS3	n.d.	n.d.	n.d.	n.d.
THBS4	Rat neonatal cardiomyocytes	Stretch	↑	[72]
	Mouse heart	Pressure	↑	[72]
	Human mesenchymal stem cells	Scaffold/substrate stiffness	↑	[73]
COMP	Human mesenchymal stem cells	Scaffold/substrate stiffness	↑	[73]
DCN	Human bronchial fibroblasts from asthmatic patients	Stretch	↑	[74]
	Human bronchial fibroblasts from healthy volunteers	Stretch	↔	[74]
	Bovine periodontal ligament cells	Stretch	↑	[75]
	Human vaginal fibroblasts from patients with pelvic organ prolapse	Stretch	↓	[76]
	Human vaginal fibroblasts from patients without pelvic organ prolapse	Stretch	↔	[76]
	Mouse left ventricle	Pressure	↑	[77]
BGN	R-ASM-580 (rat aortic smooth muscle cells)	Stretch	↑	[31]
	Human vaginal fibroblasts from patients with pelvic organ prolapse	Stretch	↔	[76]
	Human vaginal fibroblasts from patients without pelvic organ prolapse	Stretch	↑	[76]
	Mouse left ventricle	Pressure	↑	[77]
LUM	R-ASM-580 (rat aortic smooth muscle cells)	Stretch	↑	[31]
	Human bronchial fibroblasts from asthmatic patients	Stretch	↔	[74]
	Human bronchial fibroblasts from healthy volunteers	Stretch	↔	[74]
	Human vaginal fibroblasts from patients with pelvic organ prolapse	Stretch	↔	[76]
	Human vaginal fibroblasts from patients without pelvic organ prolapse	Stretch	↔	[76]
	Rat neonatal cardiac fibroblasts	Stretch	↑	[77]
	Human periodontal ligament stem cells	Stretch	↑	[78]
	Mouse left ventricle	Pressure	↑	[77]
	Mice diaphragm	Pressure	↑	[79]
ASPN	Human periodontal ligament cells	Hypergravity	↑	[80]

Abbreviations: ↔ = No change, ↑ = Upregulation, ↓ = Downregulation, n.d. = No data found.

**Table 2:** Effect of MCPs on collagen fiber architecture.

Protein	Experimental Model	Cells/Organ	Observed Effect	Source
POSTN	KO mice	Skin	Fibril size ↓	[87]
	KO mice	Tendon	Crosslinking ↓	[87]
	KO mice	Tail tendon	Fibril packing density ↓ Fibril size over 300 nm ↑	[88]
	KO mice	Calvarial osteoblasts	Crosslinking ↓ (Active LOX ↓)	[89]
	KO mice	Heart after acute myocardial infarction	Fiber density ↓ Fibril size ↓ Crosslinking ↓	[90]
	Overexpressing cells	C3H10T1/2 cells	Crosslinking ↑ (Active LOX ↑)	[89]
TGFBI	KO mice	Cornea	Fibril size ↔ Fibril packing density ↓	[93]
	Pancreatic cancer model mice treated with antibody	Pancreatic cancer	Mature fibers ↓	[91]
	Collagen matrix with recombinant protein	Scaffold	Fiber size ↑ Fiber density ↑	[92]
TNC	KO mice	Heart after acute myocardial infarction	Fiber density↓	[94]
	Correlation between ECM proteins and collagen fiber alignment	Breast cancer tissue	Correlated with fiber alignment	[95]
TNXB	KO mice	Skin	Fibril packing density ↓	[96]
	KO mice	Skin	Fibril size ↑ Fibril packing density ↔	[97]
	Collagen matrix with recombinant protein	Scaffold	Fibril size ↔	[98]
TNR	n.d.	n.d.	n.d.	n.d.
TNN	n.d.	n.d.	n.d.	n.d.
SPARC	KO mice	Iris	Fibril size ↓	[99]
	KO mice	PAN02 orthotopic tumor	Mature fibers ↓ Fiber density ↓	[100]
	KO mice	Tibialis anterior muscle	Fiber density ↓	[101]
	KO mice	Achilles tendon	Fibril size ↓ Fibril packing density ↑ Fiber alignment ↑	[102]
	KO mice	Skin	Fiber density ↓ Fibril size ↓ Mature fibers ↓	[103]
SPARCL1	KO mice	Iris	Fibril size ↔	[99]
	KO mice	Skin	Fiber density ↑ Fibril size ↓ Fibril packing density ↑	[104]
SMOC1	n.d.	n.d.	n.d.	n.d.
SMOC2	n.d.	n.d.	n.d.	n.d.
SPOCK1	Knockdown cells	Pancreatic cancer cells coculture with knockdown mouse embryonic fibroblasts	Fiber density ↓	[105]
	Overexpressing mice	Gingiva	Fiber density ↑	[106]
SPOCK2-3	n.d.	n.d.	n.d.	n.d.
CCN1	Fibroblast-specific KO mice	Heart after acute myocardial infarction	Fiber size ↔ Fiber alignment ↓ Fiber tortuosity ↓ Fibril size ↓ Fibril packing density ↓	[107]
	Fibroblast-specific KO mice	Skin	Fiber alignment ↓ Fibril size ↓ Fibril packing density ↓	[108]
CCN2	Myofibroblast-specific KO mice	Quadriceps muscle	Fiber size ↔	[109]
	Skeletal myocyte-specific KO mice	Quadriceps muscle	Fiber size ↔	[109]
	Skeletal myocyte-specific KO in muscular dystrophy mice model	Quadriceps muscle	Mature fibers ↓	[109]
	Overexpressing mice	Tibialis anterior muscle	Fiber size ↓ Mature fibers ↑	[109]
	Controlled release gels	Bovine meniscus explants	Fiber alignment ↑ (in high CCN2 dose combined with slow TGFB3 release model)	[110]
	Knockdown nanocomplexes	Rabbit ear skin after wound	Fibril size ↑ Fiber alignment ↓	[111]
	Controlled release sutures	Canine flexor digitorum profundus tendon after surgery	Fibril size ↓	[112]
CCN3	Exon 3 KO mice	Articular cartilage of knee joint	Mature fibers ↑ (in males) Mature fibers ↔ (in females)	[113]
	KO mice	Kidney after ureteral obstruction	Fiber density ↓	[114]
CCN4	Collagen matrix with recombinant protein	Scaffold	Fibril size ↔ Fibril alignment ↑	[115]
	Effect of condition media from knockdown cells on collagen matrix	Scaffold	Fibril size ↔ Fibril alignment ↔	[115]
	Overexpressing cells	4T1 orthotopic tumor	Fiber size ↑ Mature fibers ↑	[115]
	Overexpressing cells	MDA-MB-231 orthotopic tumor	Mature fibers ↑	[116]
	Knockdown cells	4T1 orthotopic tumor	Fiber size ↓ Mature fibers ↓	[115]
CCN5	Collagen matrix with recombinant protein	Scaffold	Inhibit effect of CCN4 on fiber alignment	[116]
	Overexpressing mice	4T1 and MDA-MB-231 orthotopic tumors	Mature fibers ↓	[116]
	Mice treated with recombinant protein	4T1 orthotopic tumor	Mature fibers ↓	[116]
CCN6	n.d.	n.d.	n.d.	n.d.
THBS1	Correlation between ECM proteins and collagen fiber alignment	Breast cancer tissue	Correlated with fiber alignment	[95]
	KO mice	Skin	Crosslinking ↓	[117]
	KO mice	Skin and tail tendon	Fibril size ↑ Disordered fibril packing	[118]
	KO mice	Juxtacanalicular tissue	Fibril size ↑	[119]
THBS2	Correlation between ECM proteins and collagen fiber alignment	Breast cancer tissue	Correlated with fiber alignment	[95]
	KO mice	Juxtacanalicular tissue	Fibril size ↑	[119]
	KO mice	Skin and tail tendon	Fibril size ↑ Disordered fibril packing	[120]
	KO mice	Anterior and posterior femoral middiaphysis	Fiber density ↓	[121]
	KO mice	Primary marrow-derived mesenchymal stromal cells	Mature fibers ↓	[122]
	KO mice	Primary dermal fibroblasts	Crosslinking ↓ (LOX ↓)	[123]
	KO mice	Skin after wound	Crosslinking ↓ (LOX ↓)	[123]
THBS3	n.d.	n.d.	n.d.	n.d.
THBS4	KO mice	Patellar tendon	Fibril size ↑	[124]
	KO mice	Heart after pressure overload	Fiber density ↑	[125]
	KO mice	ANG II-treated mesenteric arteries	Fiber size ↑	[126]
COMP	Overexpressing cells	Bovine chondrocytes in alginate 3D culture	Fibril size ↓	[127]
DCN	KO mice	Skin	Fibril size ↔ Disordered fibril packing	[128]
	KO mice	Tail tendon	Fibril size ↑	[128]
	KO mice	Cornea	Fibril size ↔ Normal packing	[128]
	KO mice	Cornea after wound	Fibril size ↑ Fibril packing density ↓ Disordered fibril packing	[130]
	KO mice	Skin and tail tendon	Fibril size ↑ Disordered fibril packing	[134]
	KO mice	Bone	Fibril size ↓ Disordered fibril packing	[134]
	Conditional KO mice	Patellar tendons	Fibril size ↑ Fibril packing density ↓	[133]
	Collagen matrix with purified protein	Scaffold	Fibril size ↓ Aggregation of fibrils into fibers ↓	[129]
	Collagen matrix with recombinant protein	Scaffold	Fiber size↔ Fibril size ↓	[132]
	Collagen matrix with recombinant protein	Scaffold	Fiber size ↓	[140]
DCN/BGN	Conditional double KO mice	Patellar tendons	Fibril size ↑ Fibril packing density ↓	[131]
	Double KO mice	Skin	Fibril packing density ↓ Disordered fibril packing	[134]
BGN	KO mice	Skin	Fibril size ↑ Disordered fibril packing	[134]
	KO mice	Tail tendon	Fibril size ↓ Disordered fibril packing	[134]
	KO mice	Bone	Fibril size ↑ Disordered fibril packing	[134]
	Conditional KO mice	Patellar tendons	Fibril size ↑ Fibril packing density ↓	[133]
	Collagen matrix with recombinant protein	Scaffold	Fiber size↑ Fibril size ↓	[132]
LUM	KO mice	Hind limb flexor tendon	Fibril size ↑ (4 days old mice) Fibril size ↓ (10 days old mice) Fibril size ↔ (1–3 months old mice)	[135]
	KO mice	Cornea (Posterior stroma)	Fibril size ↑ Fibril packing density ↓ Disordered fibril packing	[136]
	KO mice	Skin and cornea	Fibril size ↑ Fibril packing density ↓ Disordered fibril packing	[137]
	Collagen matrix with recombinant protein	Scaffold	Fiber size ↔	[140]
ASPN	KO Mice	Skin	Fibril size ↔	[138]
	KO Mice	Periodontal ligament	Fibril size ↑	[139]

Abbreviations: ↔ = No change, ↑ = Increased, ↓ = Decreased, n.d. = No data found.

**Table 3:** In vitro characterization of MCP-mediated effects in bladder cancer.

Protein	Experimental Model	Proliferation/Growth	Invasion/Migration	Source
POSTN	T24 cells + retrovirus vector (full-length POSTN)	↔	n.d.	[158]
	SBT31A cells + retrovirus vector (full-length POSTN)	↔	↓	[159]
	SBT31A cells + retrovirus vector (POSTN without region in N-term)	n.d.	↓	[159]
	SBT31A cells + retrovirus vector (POSTN without region in C-term)	n.d.	↓	[159]
	SBT31A cells + retrovirus vector (POSTN without regions in N- and C-term)	n.d.	↔	[159]
	SBT31A cells + retrovirus vector (POSTN C-term)	n.d.	↓	[159]
	SBT991 cells + retrovirus vector (full-length POSTN)	↔	↓	[160]
	SBT991 cells + retrovirus vector (POSTN with loss exons 17, 18, and 21)	↔	↔	[160]
	SBT991 cells + retrovirus vector (POSTN with loss exons 17 and 21)	↔	↓	[160]
	SBT31A cells + retrovirus vector (full-length POSTN)	n.d.	↓	[161]
	SBT31A cells + retrovirus vector (POSTN C-term)	n.d.	↓	[161]
	T24 cells + retrovirus vector (full-length POSTN)	n.d.	↓	[161]
	UMUC3 cells + retrovirus vector (full-length POSTN)	↔	↓	[162]
	UMUC3 cells + rPOSTN (100 ng/mL)	↔	↓	[162]
	Knockdown cells (TCC-SUP, J82)	n.d.	↓	[151]
	T24, TCC-SUP, J82 cells + rPOSTN (2 *μ*g/mL)	n.d.	↑	[151]
	T24 cells + POSTN knockdown CAFs	n.d.	↓	[163]
TGFBI	Knockdown cells (5637)	↓	↓	[152]
	Knockdown cells (RT112, 253J)	↓	↓	[164]
	Overexpressing cells (RT112, 253J)	↑	↑	[164]
	Overexpressing cells (T24, UMUC3)	n.d.	↑	[165]
	Knockdown cells (5637, T24)	↓	↓	[166]
	Overexpressing cells (5637, T24)	↑	↑	[166]
TNC	Knockdown cells (J82, T24)	↓	↓	[167]
	Overexpressing cells (5637, 253J)	↑	↑	[167]
TNXB	n.d.	n.d.	n.d.	n.d.
TNR	n.d.	n.d.	n.d.	n.d.
TNN	n.d.	n.d.	n.d.	n.d.
SPARC	Primary cancerous urothelial cells from SPARC KO mice	↑	↑	[168]
	Knockdown cells (UMUC3, T24)	↑	n.d.	[168]
	Overexpressing cells (UMUC3, T24T)	↓	n.d.	[168]
	T24M cells + antibody against SPARC	n.d.	↓	[169]
SPARCL1	Knockdown cells (EJ, T24)	↑	↑	[170]
	Overexpressing cells (EJ, T24)	↓	↓	[170]
SMOC1-2	n.d.	n.d.	n.d.	n.d.
SPOCK1-3	n.d.	n.d.	n.d.	n.d.
CCN1	n.d.	n.d.	n.d.	n.d
CCN2	Knockdown cells (5637, T24)	↓	↓	[171]
CCN3	EJ cells + retrovirus vector (truncated CCN3)	↔	↑	[172]
CCN4	HT1376 and T24 cells + rCCN4 (400 ng/mL)	↑	n.d.	[173]
	CCN4 isoform 1 overexpressing cells (HT1376)	↑	↑	[173]
	CCN4 isoform 1 overexpressing cells (T24)	n.d.	↑	[173]
	CCN4 isoform 2 overexpressing cells (HT1376)	↔	↔	[173]
CCN5-6	n.d.	n.d.	n.d.	n.d.
THBS1-3	n.d.	n.d.	n.d.	n.d.
THBS4	T24 + rTHBS4 (3–30 ng/mL)	n.d.	↑	[174]
COMP	n.d.	n.d.	n.d.	n.d.
DCN	T24 + adenovirus vector (DCN)	↓	n.d.	[175]
	Knockdown cells (MB49-I)	↔	n.d.	[176]
	Knockdown cells (TCC-SUP)	n.d.	↓	[176]
	Knockdown cancer stem cells (SW780)	↓	n.d.	[177]
	T24 + retrovirus vector (full-length DCN)	↓	↓	[177]
	T24 + retrovirus vector (non-glycanated DCN)	↑	↑	[177]
BGN	J82 + rBGN (0.26–26 nM)	↓	n.d.	[178]
	Knockdown cells (J82)	↑	n.d.	[178]
	Overexpressing cells (J82)	↓	n.d.	[178]
	Knockdown cells (J82) + rBGN	↔	n.d.	[178]
LUM	Knockdown cells (T24, J82)	↓	↓	[179]
ASPN	Knockdown cells (5637, J82)	n.d.	↓	[180]

Abbreviations: ↔ = No change, ↑ = Increased, ↓ = Decreased, n.d. = No data found.

**Table 4:** In vivo characterization of MCP-mediated effects in bladder cancer.

Protein	Experimental Model	Mice	Tumor Weight/Size	Metastases	Source
POSTN	Subcutaneous injection of UMUC3 cells + retrovirus vector (full-length POSTN)	BALB/c nude	↔	n.d.	[162]
	Orthotopic implantation of UMUC3 cells + retrovirus vector (full-length POSTN)	BALB/c nude	↓ *	n.d.	[162]
TGFBI	Subcutaneous injection of Overexpressing cells (5367)	BALB/c nude	↑	n.d.	[166]
SPARC	Subcutaneous injection of MB49	SPARC KO C57BL/6	↑	?	[168]
	Subcutaneous injection of knockdown cells (UMUC3, T24)	Athymic nude	↑	n.d.	[168]
	Subcutaneous injection of overexpressing cells (UMUC3, T24T)	Athymic nude	↓	n.d.	[168]
	Intravenous injection of knockdown cells (UMUC3, T24)	Athymic nude	n.d.	↑	[168]
	Intravenous injection of overexpressing cells (UMUC3, T24T)	Athymic nude	n.d.	↓	[168]
CCN2	Subcutaneous injection of knockdown cells (T24)	BALB/c nude	↓	n.d.	[171]
CCN4	Subcutaneous injection of CCN4 isoform 1 overexpressing cells (HT1376)	BALB/c nude	↑	n.d.	[173]
DCN	Subcutaneous injection of overexpressing cells (MB49)	C57Bl/6J	↑	n.d.	[176]
	Orthotopic implantation of knockdown cells (MB49-I)	C57Bl/6J	↓ *	n.d.	[176]
	Subcutaneous injection of T24 + retrovirus vector (full-length DCN)	BALB/c nude	↓	n.d.	[177]
	Subcutaneous injection of T24 + retrovirus vector (non-glycanated DCN)	BALB/c nude	↑	n.d.	[177]
BGN	Subcutaneous injection of Knockdown cells (J82)	NMRI nu/nu	↑	n.d.	[178]
LUM	Subcutaneous injection of Knockdown cells (T24)	BALB/c nude	↓	n.d.	[179]

Abbreviations: ↔ = No statistically significant change, ↑ = Increased, ↓ = Decreased, * = Bladder weight with tumor, n.d. = No data found, ? = Mentioned in the article without quantitative data or statistical comparison.

## Data Availability

Publicly available datasets were analyzed in this study. The TCGA-BLCA dataset [187] is available from the Genomic Data Commons at https://portal.gdc.cancer.gov/projects/TCGA-BLCA (accessed on 19 March 2026). The Genotype-Tissue Expression (GTEx) v11 data [188] are available from https://gtexportal.org/ (accessed on 19 March 2026). Cancer Cell Line Encyclopedia (CCLE) data [189] are available from the DepMap portal at https://depmap.org/ (accessed on 30 April 2026). The GSE167430 [86] and GSE47080 [85] datasets are available from the Gene Expression Omnibus at https://www.ncbi.nlm.nih.gov/geo/ (accessed on 23 June 2026). The SCP3121 dataset [147] is available from the Single Cell Portal at https://singlecell.broadinstitute.org/single_cell (accessed on 2 April 2026). The code used for data analysis is publicly available on GitHub at https://github.com/Alim220v/mechanoMCPs (accessed on 2 August 2026). Data sharing does not apply to this article, as no new data were created.

## Abbreviations

The following abbreviations are used in this manuscript:

ANG II	Angiotensin II
ASPN	Asporin
BBN	N-butyl-N-(4-hydroxybutyl)-nitrosamine
BGN	Biglycan
BLCA	Bladder Urothelial Carcinoma
BOO	Bladder Outlet Obstruction
CAFs	Cancer-Associated Fibroblasts
CCLE	Cancer Cell Line Encyclopedia
CCN	Cellular Communication Network Factor
COL1A1	Collagen Type I Alpha 1 Chain
COL3A1	Collagen Type III Alpha 1 Chain
COMP	Cartilage Oligomeric Matrix Protein
DCN	Decorin
ECM	Extracellular Matrix
GTEx	The Genotype-Tissue Expression Portal
KO	Knockout
LOX	Lysyl Oxidase
LUM	Lumican
MCPs	Matricellular Proteins
MNU	N-methyl-N-nitrosourea
POSTN	Periostin
r	Recombinant (prefix for recombinant proteins)
SMOC	SPARC-Related Modular Calcium-Binding Protein
SPARC	Secreted Protein Acidic and Rich in Cysteine Protein
SPARCL1	SPARC-like protein 1
SPOCK	SPARC/Osteonectin, CWCV and Kazal-Like Domains Proteoglycan
TCGA	The Cancer Genome Atlas
TGFB3	Transforming Growth Factor Beta 3
TGFBI	Transforming Growth Factor Beta-Induced Protein
THBS	Thrombospondin
TNC	Tenascin C
TNN	Tenascin N
TNR	Tenascin R
TNXB	Tenascin X
TPM	Transcripts Per Million
VEGF-C	Vascular Endothelial Growth Factor C

## Appendix A. Materials and Methods

### Appendix A.1. Protein-Protein Interaction Network

The protein–protein interaction network was constructed using the STRING database (version 12.0, https://string-db.org/, accessed on 22 July 2026) [190] with a minimum required interaction score of 0.4 (medium confidence) and visualized in confidence view.

### Appendix A.2. MCPs Expression in Bulk RNA-Seq

GTEx (normal tissue) [188] and TCGA-BLCA (tumor and adjacent normal) [187] RNA-seq datasets were used in this study. For the TCGA-BLCA cohort, samples lacking AJCC pathological stage information (TCGA-FJ-A3Z9 and TCGA-HQ-A2OE) were excluded. Remaining TCGA-BLCA samples were classified as tumor or adjacent normal tissue according to standard TCGA barcode sample type codes (codes 01–09 for primary tumor; codes 10–19 for adjacent normal). For patients with multiple tumor RNA-seq samples, gene expression values were averaged to obtain a single TPM profile per patient. A heatmap was generated using $\log_{2}$(TPM + 1) values across the combined GTEx and TCGA-BLCA samples. Stromal scores for TCGA-BLCA were obtained from pre-computed ESTIMATE scores [191] available at https://bioinformatics.mdanderson.org/estimate (accessed on 9 April 2026).

### Appendix A.3. Differential Expression Analysis of Stiffness-Stratified Tumors

Tumor samples from the TCGA-BLCA dataset [187] were analyzed. Duplicate samples were removed, and only protein-coding genes were retained. Genes with counts ≥10 in fewer than 150 samples were excluded. Tumors were stratified into stiff and soft groups based on the expression of three matrix stiffness-associated genes (COL1A1, LOX, and COL3A1). Samples in the upper quartile (≥Q75) for all three genes were classified as stiff (*n* = 70), while samples in the lower quartile (≤Q25) for all three genes were classified as soft (*n* = 70). Differential expression analysis was performed using PyDESeq2 v0.5.0 [192] and an adjusted *p*-value threshold of <0.05 and an absolute log2 fold change threshold of >0.5 was applied.

### Appendix A.4. MCPs Expression in Bladder Cell Lines

RNA-seq expression data and cell-line annotations were obtained from the Cancer Cell Line Encyclopedia [189]. Bladder cell lines were identified by filtering the annotation table. Two lines absent from the expression matrix (RT11284 and ACCS) were excluded. A heatmap was generated to visualize $\log_{2}$(TPM + 1) values across the selected cell lines.

### Appendix A.5. Differential Expression Analysis of MCPs in Rat Bladder Partial Outlet Obstruction Datasets

The GSE47080 dataset [85] was analyzed using GEO2R [193] on the GPL6247 platform. Differential expression analysis was performed to compare the control group (*n* = 6) with the 10-day (*n* = 6) and 6-week (*n* = 6) obstruction groups. Differentially expressed genes were identified using an adjusted *p*-value threshold of <0.05 and an absolute $log_{2}$ fold change threshold of >0.5.

The GSE167430 dataset [86] was analyzed using the European Galaxy server (https://usegalaxy.eu/, accessed on 23 June 2026) [194]. Raw RNA-seq data were processed with Trimmomatic v0.39 [195] for adapter trimming and quality filtering. Reads were aligned to the rat genome (rn7) using HISAT2 v2.2.2 [196], and transcript assembly was performed with StringTie v2.2.3 [197]. Differential expression analysis between the control (*n* = 3) and 4-week obstruction group (*n* = 3) was then performed locally using PyDESeq2 v0.5.0 [192] and an adjusted *p*-value threshold of <0.05 and an absolute $log_{2}$ fold change threshold of >0.5 was applied.

### Appendix A.6. MCPs Expression in Single Cell RNA-Seq

A single-cell RNA-seq dataset [147] containing bladder cancer patients [145] and normal bladder samples [198] was filtered to genes encoding matricellular proteins. Several MCP genes (CCN1–6, TNR, TNN, and SPOCK3) were absent in the dataset. The remaining genes were visualized using dot plots of mean expression across cell clusters with Scanpy v1.11.5 [199]. Differential expression between CAFs and normal fibroblasts was assessed using the Wilcoxon rank-sum test, and results were filtered to MCP genes with an adjusted *p*-value < 0.05.

### Appendix A.7. Survival Analysis

Survival analysis of matricellular protein gene expression was conducted on RNA-seq and clinical data from bladder cancer patients in TCGA-BLCA [187] using the lifelines v0.30 [200]. For each of the 28 MCP genes, patients were dichotomized into high-expression (≥Q75, *n* = 100) and low/medium-expression groups (*n* = 300). Time-to-event was defined as days to death for deceased patients and days to the last follow-up for living patients. Kaplan–Meier survival curves were generated for each group, differences between groups were tested with the log-rank test, and hazard ratios with 95% confidence intervals were estimated using univariate Cox proportional hazards models. Log-rank *p*-values were adjusted for multiple testing across the 28 genes using the Benjamini–Hochberg false discovery rate (FDR) procedure, and genes with FDR < 0.05 were considered statistically significant. Results were visualized in a volcano plot of $log_{2}$ (hazard ratio) versus $-log_{10}$ (log-rank *p*-value).
